# Days Alive and Out of Hospital at 15 Days after Hip Replacement May Be Associated with Long-Term Mortality: Observational Cohort Study

**DOI:** 10.3390/diagnostics13061155

**Published:** 2023-03-17

**Authors:** Ah Ran Oh, Ji-Hye Kwon, Jungchan Park, Gayoung Jin, So Myung Kong, Sangmin Maria Lee

**Affiliations:** 1 Department of Anesthesiology and Pain Medicine, Samsung Medical Center, Sungkyunkwan University School of Medicine, Seoul 06351, Republic of Korea; 2 Department of Anesthesiology and Pain Medicine, Kangwon National University Hospital, Chuncheon 24341, Republic of Korea

**Keywords:** outcome, mortality, hip replacement

## Abstract

We aimed to evaluate the association between days alive and out of hospital (DAOH) and mortality at 15 days after a hip replacement. From March 2010 to June 2020, we identified 5369 consecutive adult patients undergoing hip replacements and estimated DAOH at 15, 30, 60, and 90 days after surgery. After excluding 13 patients who died within 15 days after surgery, receiver operating characteristic (ROC) curves were then generated to evaluate predictabilities for each follow-up period. We compared the mortality risk according to the estimated thresholds of DAOH at 15 days after hip replacement. ROC analysis revealed areas under the curve of 0.862, 0.877, 0.906, and 0.922 for DAOH at 15, 30, 60, and 90 days after surgery, respectively. The estimated threshold of DAOH during the 15 postoperative days was 6.5. Patients were divided according to this threshold, and propensity score matching was conducted. In a propensity score-matched population with 864 patients in each group, the risk of mortality increased in patients with a lower DAOH 15 (2.8% vs. 8.1%; hazard ratio [HR] = 3.96; 95% confidence interval [CI]: 2.24–6.99; *p* < 0.001 for one-year mortality, 5.2% vs. 13.0%; HR = 3.82; 95% CI: 2.33–6.28; *p* < 0.001 for three-year mortality, and 5.9% vs. 15.6%; HR = 3.07; 95% CI: 2.04–4.61; *p* < 0.001 for five-year mortality). In patients undergoing a hip replacement, DAOH at 15 days after surgery was shown to be associated with increased mortality. DAOH at 15 days may be used as a valid outcome measure for hip replacement.

## 1. Introduction

Hip replacement surgery is a common and effective procedure for treating various hip conditions, including hip fractures and osteoarthritis [1]. With an increase in life expectancy and aging population, the demand for hip replacement surgery has continued to rise. While hip replacement surgery is generally considered safe, the mortality rate following the procedure remains relatively high, with some studies reporting rates as high as 0.5% within the first year after surgery [2,3]. Therefore, it is essential to conduct studies on the outcomes of hip replacement surgery and to identify effective outcome measures that can accurately reflect long-term survival. This is a crucial area of research because the long-term survival of patients undergoing hip replacement surgery is closely related to their overall well-being and quality of life. In addition to mortality rates, postoperative complications can have a significant impact on a patient’s quality of life and long-term survival. Thus, it is essential to conduct studies on the outcomes of hip replacement surgery and to identify effective outcome measures that can accurately reflect long-term survival.

Days Alive and Out of Hospital (DAOH) is a relatively new outcome measure that has gained popularity due to its simplicity and ease of calculation using readily available variables [4]. It was initially introduced to evaluate the outcomes of patients with chronic diseases but has since been validated in acute diseases [5] and various surgical procedures [4,6,7,8]. The measure calculates the time a patient is alive and out of the hospital, which reflects the overall well-being of the patient as well as the efficiency of the healthcare system. The use of DAOH is particularly relevant in hip replacement surgery because it provides an objective measure of the recovery period after the procedure.

The Standardized Endpoints in Perioperative Medicine (StEP) initiative has recommended DAOH after surgery as a reliable outcome measure, emphasizing its potential to reflect patient-centered outcomes [9]. The strength of DAOH is that it can be determined by subtracting the total days of an initial or subsequent in-hospital stay from the total length of the period, providing an objective measure of the time a patient spends alive and out of the hospital. However, determining the optimal follow-up duration for DAOH in predicting long-term outcomes after surgery is a significant challenge. While a longer follow-up period would result in a stronger correlation with outcomes, a shorter follow-up period would enable DAOH to be obtained more quickly for more patients. The optimal follow-up period for DAOH after hip replacement surgery remains unclear, and this study aims to address this gap by analyzing the correlation between DAOH at different time points (15, 30, 60, and 90 days) and postoperative mortality. By identifying the optimal follow-up duration for DAOH in predicting long-term outcomes after hip replacement surgery, we hope to improve patient outcomes, reduce mortality rates, and optimize healthcare resource allocation following this common surgical procedure.

## 2. Materials and Methods

Our study was a retrospective observational cohort study which was conducted from 17 January 2023 to 28 February 2023. This study was conducted in accordance with the ethical principles outlined in the Declaration of Helsinki and followed the Strengthening the Reporting of Observational Studies in Epidemiology (STROBE) guidelines. As the data were collected in de-identified form and posed minimal risk to the study patients, the need for institutional review board (IRB) approval was waived at our institution on 17 January 2023 (Samsung Medical Center, 81 Irwon-ro, Gangnam-gu, Seoul, Korea, 2023-01-060). The decision to waive IRB approval was made by the chairperson, Prof. S.W. Park, based on the criteria outlined in the institutional policies and procedures.

Furthermore, we did not obtain written informed consent from individual patients, because the data used in the study were collected retrospectively from electronic medical records. To ensure patient privacy and confidentiality, all personal identifying information was removed from the dataset before analysis. The use of de-identified data minimized the potential risks to study participants, and we took appropriate measures to ensure data security and confidentiality throughout the study.

We also followed the STROBE guidelines for reporting observational studies, which require a comprehensive and transparent reporting of study methods and results to improve the quality and reliability of scientific research. By adhering to these guidelines, we aim to promote scientific rigor and transparency when reporting the findings of our study, as well as facilitate the replication and extension of our research by other investigators.

### 2.1. Study Population and Data Collection

We used the electronic medical records from the Samsung Medical Center, a tertiary referral hospital in Seoul, Korea, between March 2010 and June 2020. Our search criteria were limited to adult patients who underwent hip replacement surgery during this period. To ensure the privacy and confidentiality of the patients, the data were collected in a de-identified form using the “Clinical Data Warehouse Darwin-C” electronic archive system. This system enables the retrieval of data from electronic hospital records, including over 2.2 million surgeries, 1 billion laboratory results, 100 million disease codes, and 200 million prescriptions.

Blood test results were automatically processed, which ensured the accuracy and consistency of the data collected. Mortality data were regularly checked and updated using the National Population Registry of the Korea National Statistical Office to ensure completeness. Medical records were reviewed by investigators who were not informed of patient mortality to prevent bias. In addition, we excluded patients who had died within 15 days after surgery from our analysis as their mortality could not be accurately attributed to the hip replacement surgery and might have introduced bias to our results.

Overall, our study used a comprehensive and reliable database to identify eligible patients and collect data on important clinical outcomes. By using a large-scale electronic medical records system, we were able to collect data efficiently and accurately, which improved the validity and generalizability of our findings.

### 2.2. Study Outcomes and Definitions

The primary endpoint of this study was to assess the relationship between one-year mortality and DAOH at 15, 30, 60, and 90 days after hip replacement surgery. Additionally, the relationship between DAOH and mortality during three- and five-year follow-up periods was also examined to investigate any potential long-term effects.

DAOH was calculated using the same method as previously described [4]. Specifically, it was determined by subtracting the total duration of an initial or subsequent in-hospital stay from the defined time periods of 15, 30, 60, and 90 days. If a patient died within the defined period, DAOH was recorded as 0, reflecting that the patient was not alive and out of the hospital during that time. Thus, the DAOH value ranged from 0 to the defined time period, with a lower number indicating a poorer outcome.

To assess the baseline health status of the patients, the Charlson Comorbidity Index was calculated using the 10th revision of the International Statistical Classification of Diseases and Related Health Problems (ICD-10) [10]. The Charlson Comorbidity Index is a widely used tool to assess comorbid conditions and predict the risk of mortality. It takes into account the presence of 19 different comorbid conditions, each of which is assigned a weighted score. The sum of the scores yields the total Charlson Comorbidity Index score, with a higher score indicating a greater number and severity of comorbidities. The Charlson Comorbidity Index was used in this study to adjust for potential confounding factors that could affect the relationship between DAOH and mortality.

### 2.3. Statistical Analysis

In this study, categorical variables were presented as count and percentage, and continuous variables were represented as mean and standard deviation or median and interquartile range (IQR) based on the appropriate measure of central tendency. Comparison of categorical variables was performed using a chi-square test, and continuous variables were compared using a *t*-test or Mann–Whitney test. The receiver operating characteristic (ROC) curve analysis was conducted to evaluate the correlation between DAOH and mortality, and Youden’s Index was utilized to determine the optimal cut-off point. A comparison of ROC curves was made using the DeLong test [11]. After dividing patients into low and high groups based on the calculated cut-off points, the mortality rates were compared using a Cox regression analysis. The results were reported as hazard ratios (HRs) with 95% confidence intervals (CIs). To evaluate the effect of demographic factors on the outcomes of interest, a subgroup analysis was conducted based on age (older or younger than 60 years), sex, type of anesthesia (general or regional), indication for surgery (emergency or elective), and total hip replacement. The demographic and baseline characteristics of each subgroup are presented as a forest plot. To further reduce bias and achieve balance between groups, propensity score matching was conducted using a 0.25 caliper. The matching process created a 1:1 matched population by pairing patients with similar preoperative characteristics to minimize the effect of confounding variables on the outcome of interest (mortality). The balance between groups was deemed successful if the absolute standardized difference (ASD) was less than 10%. All statistical analyses were performed using R version 4.2.0, and a *p*-value less than 0.05 was considered statistically significant.

## 3. Results

This study included 5369 adult patients who underwent hip replacement surgery between March 2010 and June 2020 at Samsung Medical Center. Thirteen patients who died within 15 days after surgery were excluded, leaving a total of 5356 patients. The median age of the patients was 60 years old (interquartile range: 48–72). Among them, 86.4% (4628/5356) underwent a total hip replacement.

**Figure 1:**
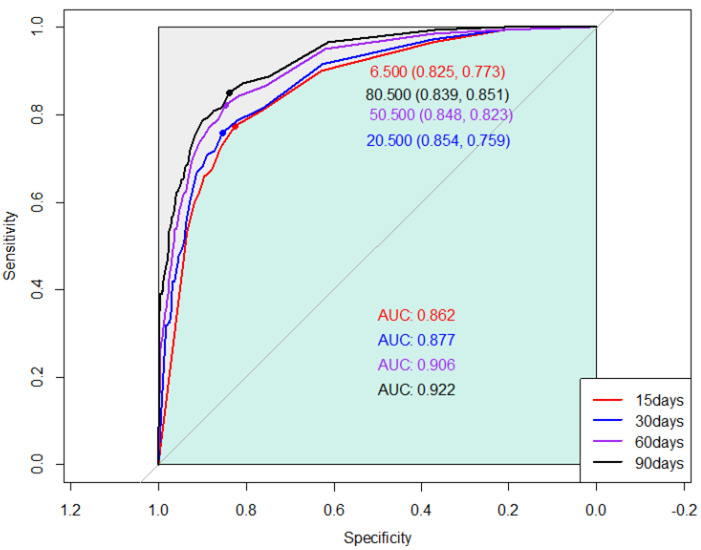
Receiver operating curves showing the association between one-year mortality and days alive and out of hospital (DAOH) at 15, 30, 60, and 90 days after hip replacement surgery.

Receiver operating characteristic (ROC) curves were generated to assess the correlation between days alive and out of hospital (DAOH) and mortality during each follow-up duration (15, 30, 60, and 90 days). The area under the curve (AUC) for DAOH at 15, 30, 60, and 90 days were 0.862, 0.877, 0.906, and 0.922, respectively. The AUCs were significantly different between DAOH at 15 days and those of other follow-up durations. The optimal cut-off threshold value for DAOH at 30 days, as determined by the maximum Youden’s Index, was 6.5 days (Figure 1).

The readmission rate within 15 days after surgery was 0.6% (30/5356). The quartile values for DAOH at 15 postoperative days were 7, 9, and 10, respectively, and patients were divided accordingly. Table 1 presents the baseline characteristics and mortality of patients. Patients with a higher DAOH at 15 days postoperative tended to have a higher DAOH over longer follow-up periods and lower risk of mortality.

The optimal cut-off value of DAOH at 15 postoperative days was estimated to be 6.5 days, and patients were classified at 7 days: 4334 (80.9%) in the high and 1022 (19.1%) in the low groups. Baseline characteristics are summarized in Table 2. The low group showed an increased risk of mortality (0.7% vs. 10.7%; HR = 14.90; 95% CI: 10.05–22.10; *p* < 0.001 for the one-year follow-up; 1.5% vs. 16.4%; HR = 10.71; 95% CI: 8.07–14.22; *p* < 0.001 for the three-year follow-up; and 2.0% vs. 19.5%; HR = 9.42; 95% CI: 7.32–12.12; *p* < 0.001 for the five-year follow-up; Table 3).

After propensity score matching, 864 study population pairs were generated, and an ASD less than 10% suggested well-balanced covariates between the groups. The association between DAOH at 15 postoperative days and mortalities persisted in the propensity score-matched population (2.8% vs. 8.1%; HR = 3.96; 95% CI: 2.24–6.99; *p* < 0.001 for one-year mortality; 5.2% vs. 13.0%; HR = 3.82; 95% CI: 2.33–6.28; *p* < 0.001 for three-year mortality; and 5.9% vs. 15.6%; HR = 3.07; 95% CI: 2.04–4.61; *p* < 0.001 for five-year mortality; Table 3). The results of the subgroup analysis showed that the association between DAOH and mortality was significant regardless of the demographic factors considered, including age (older or younger than 60 years), sex, type of anesthesia, indication for surgery, and total hip replacement (Table 4).

## 4. Discussion

The results of our study indicate a strong correlation between DAOH and long-term mortality after hip replacement surgery. This relationship was also apparent for DAOH calculated 15 days after surgery. This information can be used to improve patient care by identifying patients who are at higher risk of mortality, and in taking steps to reduce that risk. Overall, these findings are significant because they provide a new way to assess patient outcomes following hip replacement surgery. By using DAOH as a measure of success, clinicians can better understand the long-term impact of the surgery and identify areas for improvement.

An objective and standardized method for evaluating outcomes is of paramount importance in the field of medicine, particularly in clinical trials and quality improvement programs [9]. Traditional measures, such as hospital length of stay, have limitations because they do not fully encompass early mortality and fail to provide a comprehensive evaluation of patient outcomes. Therefore, the need for a reliable and objective measure of outcome that extends beyond the postoperative recovery period has been recognized. DAOH is a newer concept that has emerged as a potential solution to this issue [4,9]. It offers several advantages over traditional outcome measures because it does not necessitate the evaluation of individual events and has been demonstrated to be a reliable predictor of long-term mortality in a diverse patient population. In the context of surgical procedures, DAOH has particular importance as an outcome measure because it does not necessitate the evaluation of individual events. DAOH was initially introduced in 2017 [4] as a measure of perioperative outcomes and has since been confirmed through studies in diverse patient populations, including Swedish surgical patients [12], Canadian patients undergoing elective surgery [8], Danish patients undergoing hip and knee arthroplasties [13], and English patients undergoing emergency laparotomies [7]. As a result, it has been recognized as a comprehensive outcome measure that effectively reflects perioperative risks and complications. In addition, it can be easily calculated and incorporated into daily practice. Furthermore, an advantage of DAOH as a measure of outcomes is that it encompasses multiple cardiovascular events into a single, continuous metric which can be conveniently used in clinical trials. In summary, DAOH offers a reliable, comprehensive, and objective measure of patient outcomes, making it a valuable tool in clinical trials and quality improvement programs.

Previous studies [7,8,13] and the StEP initiative [9] have recommended using DAOH at 30 days, which has become the most widely used approach. Our study added that DAOH at 15 days can also be used for patients undergoing hip replacement surgery. In fact, an important consideration when applying DAOH is determining the duration of the follow-up period. It is expected that the correlation with outcomes would improve as the follow-up period of DAOH increases. However, using a shorter follow-up period for DAOH can make it more accessible to a larger number of patients. A previous study warned that a mortality rate over 10% can significantly impact DAOH [4] because in this scenario a DAOH value of 0 may indicate death rather than a longer hospital stay. Our results indicate that DAOH during a longer follow-up period had a significantly higher AUC value. However, even DAOH calculated 15 days after surgery had a decent predictive value for long-term follow-up.

The importance of shortening the follow-up period required to evaluate postoperative outcomes has become increasingly significant in recent decades because there has been a systematic implementation of evidence-based perioperative care protocols such as fast-track or enhanced recovery pathways [14,15]. The introduction of these pathways has led to improved postoperative outcomes, including reduced hospital stay, lower medical costs, and improved complication rates [16]. In orthopedic replacement surgery, the implementation of rapid recovery systems has been shown to decrease hospital stays from 4–10 days to 1–3 days, with about 15% of patients being eligible for outpatient surgery [17,18,19,20]. The shortened hospital stay may be closely related to our findings that DAOH can be used as an outcome measure 15 days after hip replacement surgery.

There are several limitations to consider when interpreting our study results. Firstly, being a retrospective single-center study, our results may be biased by unidentifiable factors, despite statistical adjustments. Secondly, the long study period may have introduced changes in surgical techniques and postoperative care that could impact the results. Thirdly, we estimated the optimal cut-off point for DAOH at each follow-up period, but this could be highly influenced by the institution’s clinical protocol and therefore may not be generalizable to other patient populations. The results should only be considered as indicating a relationship between DAOH and the outcomes of hip replacement surgery. Further, well-designed studies with multi-center data are required to validate our findings. Despite these limitations, this study is the first to show a correlation between postoperative outcomes of hip replacement surgery and DAOH 15 days postoperative. Our findings may be useful for related investigation and quality improvement of hip replacement surgery.

## 5. Conclusions

In conclusion, our study demonstrated a correlation between postoperative outcomes and DAOH 15 days after hip replacement surgery. Further studies are needed for DAOH at 15 days to be set as a useful outcome measure in patients undergoing hip replacement surgery.

## Figures and Tables

**Table 1:** Baseline characteristics and mortality according to quartile values of days alive and out of hospital (DAOH).

	DAOH < 7 (*n* = 1022)	7 ≤ DAOH < 9 (*n* = 1051)	9 ≤ DAOH < 10 (*n* = 1333)	10 ≤ DAOH < 13 (*n* = 1950)
DAOH at 15 days after hip replacement	2.4 (±2.4)	7.7 (±0.5)	9.0 (±0)	10.6 (±0.6)
DAOH at 30 days after hip replacement	13.7 (±7.3)	22.6 (±1.3)	24.0 (±0.7)	25.6 (±0.6)
DAOH at 60 days after hip replacement	40.7 (±13.3)	52.3 (±3.2)	53.8 (±2.2)	55.5 (±2.3)
DAOH at 90 days after hip replacement	68.1 (±19.6)	82.1 (±4.2)	83.6 (±4.3)	85.3 (±4.1)
Age, years	66.7 (±16.4)	59.8 (±16.1)	57.7 (±15.6)	56.7 (±14.5)
Male	367 (35.9)	440 (41.9)	597 (44.8)	880 (45.1)
Operative variables				
Duration, minutes	88.9 (±55.5)	78.6 (±38.7)	71.0 (±28.3)	69.2 (±29.8)
General anesthesia	362 (35.4)	166 (15.8)	150 (11.3)	118 (6.1)
Total hip surgery	584 (57.1)	916 (87.2)	1239 (92.9)	1889 (96.9)
Emergency surgery	208 (20.4)	107 (10.2)	88 (6.6)	57 (2.9)
Habitual risk factor				
Alcohol	172 (16.8)	288 (27.4)	401 (30.1)	637 (32.7)
Smoking	82 (8.0)	150 (14.3)	218 (16.4)	303 (15.5)
Charlson comorbidity index	0.80 (±1.58)	0.46 (±1.22)	0.35 (±0.98)	0.27 (±0.82)
Myocardial infarction	8 (0.8)	5 (0.5)	4 (0.3)	2 (0.1)
Heart failure	15 (1.5)	7 (0.7)	5 (0.4)	6 (0.3)
Peripheral vascular disease	6 (0.6)	3 (0.3)	3 (0.2)	4 (0.2)
Cerebrovascular disease	53 (5.2)	36 (3.4)	38 (2.9)	60 (3.1)
Dementia	0 (0.0)	1 (0.1)	0 (0.0)	0 (0.0)
Chronic pulmonary disease	3 (0.3)	0 (0.0)	0 (0.0)	0 (0.0)
Rheumatic disease	30 (2.9)	39 (3.7)	34 (2.6)	60 (3.1)
Peptic ulcer disease	0 (0.0)	1 (0.1)	1 (0.1)	1 (0.1)
Mild liver disease	90 (8.8)	56 (5.3)	71 (5.3)	79 (4.1)
Diabetes without complications	129 (12.6)	70 (6.7)	67 (5.0)	84 (4.3)
Diabetes with complications	51 (5.0)	19 (1.8)	19 (1.4)	9 (0.5)
Hemiplegia	12 (1.2)	5 (0.5)	2 (0.2)	5 (0.3)
Renal disease	83 (8.1)	52 (4.9)	55 (4.1)	50 (2.6)
Any malignancy	4 (0.4)	3 (0.3)	1 (0.1)	1 (0.1)
Moderate to severe liver disease	7 (0.7)	4 (0.4)	2 (0.2)	3 (0.2)
Metastatic solid tumor	1022 (100.0)	1051 (100.0)	1333 (100.0)	1950 (100.0)
Human immunodeficiency virus	0 (0.0)	2 (0.2)	1 (0.1)	1 (0.1)
Preoperative blood test				
Hemoglobin, g/dL	12.2 (±1.9)	13.1 (±1.7)	13.4 (±1.6)	13.5 (±1.6)
Creatinine, mg/dL	1.05 (±1.12)	0.91 (±0.81)	0.87 (±0.66)	0.28 (±0.83)
Mortality				
One-year mortality	109 (10.7)	18 (1.7)	9 (0.7)	5 (0.3)
Three-year mortality	168 (16.4)	39 (3.7)	18 (1.4)	10 (0.5)
Five-year mortality	199 (19.5)	53 (5.0)	24 (1.8)	10 (0.5)

Values are *n* (%), mean (±standardized difference), or median (interquartile range).

**Table 2:** Baseline characteristics according to days alive and out of hospital (DAOH).

	Entire Population				Propensity-Matched Population		
	High Group	Low Group	*p*-Value	ASD	High Group	Low Group	ASD
	(*n* = 4334)	(*n* = 1022)			(*n* = 864)	(*n* = 864)	
DAOH at 15 days after hip replacement	9.4 (±1.3)	2.4 (±2.4)			9.1 (±1.3)	2.6 (±2.4)	
DAOH at 30 days after hip replacement	24.4 (±1.5)	13.7 (±7.3)			23.9 (±2.0)	14.4 (±7.0)	
DAOH at 60 days after hip replacement	54.2 (±2.8)	40.7 (±13.3)			53.3 (±5.4)	41.9 (±12.3)	
DAOH at 90 days after hip replacement	84.0 (±4.4)	68.1 (±19.6)			82.8 (±8.5)	69.7 (±18.3)	
Age, years	57.7 (±15.3)	66.7 (±16.4)	<0.001	56.8	65.9 (±15.9)	65.6 (±16.5)	
Male	1917 (44.2)	367 (35.9)	<0.001	17.2	308 (35.6)	307 (35.5)	0.002
Operative variables							
Duration, minutes	72.1 (±32.0)	88.9 (±55.5)	<0.001	37.2	83.0 (±45.7)	82.8 (±43.0)	0.5
General anesthesia	434 (10.0)	362 (35.4)	<0.001	63.7	226 (26.2)	236 (27.3)	0.026
Total hip surgery	4044 (93.3)	584 (57.1)	<0.001	92.5	587 (67.9)	575 (66.6)	0.03
Emergency surgery	252 (5.8)	208 (20.4)	<0.001	44.6	167 (19.3)	160 (18.5)	0.021
Habitual risk factor							
Alcohol	1326 (30.6)	172 (16.8)	<0.001	32.7	141 (16.3)	155 (17.9)	0.043
Smoking	671 (15.5)	82 (8.0)	<0.001	23.4	69 (8.0)	73 (8.4)	0.017
Charlson comorbidity index	0.34 (±0.99)	0.80 (±1.58)	<0.001	34.1	0.66 (±1.48)	0.67 (±1.42)	0.7
Myocardial infarction	11 (0.3)	8 (0.8)	0.008	8.3	4 (0.5)	6 (0.7)	0.031
Heart failure	18 (0.4)	15 (1.5)	<0.001	11.6	9 (1.0)	7 (0.8)	0.024
Peripheral vascular disease	10 (0.2)	6 (0.6)	0.12	5.6	6 (0.7)	2 (0.2)	0.068
Cerebrovascular disease	134 (3.1)	53 (5.2)	0.002	10.3	44 (5.1)	38 (4.4)	0.033
Dementia	1 (0.0)	0 (0.0)	>0.99	2.1	1 (0.1)	0 (0.0)	0.048
Chronic pulmonary disease	0 (0.0)	3 (0.3)	0.01	7.7	864 (100.0)	864 (100.0)	<0.001
Rheumatic disease	133 (3.1)	30 (2.9)	0.91	0.3	21 (2.4)	28 (3.2)	0.049
Peptic ulcer disease	3 (0.1)	0 (0.0)	0.031	3.7	2 (0.2)	0 (0.0)	0.068
Mild liver disease	206 (4.8)	90 (8.8)	<0.001	16.2	53 (6.1)	69 (8.0)	0.072
Diabetes without complications	221 (5.1)	129 (12.6)	<0.001	26.8	88 (10.2)	96 (11.1)	0.03
Diabetes with complications	47 (1.1)	51 (5.0)	<0.001	22.3	27 (3.1)	36 (4.2)	0.056
Hemiplegia	12 (0.3)	12 (1.2)	0.001	10.2	6 (0.7)	8 (0.9)	0.026
Renal disease	157 (3.6)	83 (8.1)	0.13	19.1	72 (8.3)	60 (6.9)	0.052
Any malignancy	5 (0.1)	4 (0.4)	0.13	5.5	0 (0.0)	1 (0.1)	0.048
Moderate to severe liver disease	9 (0.2)	7 (0.7)	0.03	7.1	6 (0.7)	5 (0.6)	0.015
Metastatic solid tumor	4334 (100.0)	1022 (100.0)			864 (100.0)	864 (100.0)	<0.001
Human immunodeficiency virus	4 (0.1)	0 (0.0)	0.74	4.3	2 (0.2)	0 (0.0)	0.068
Preoperative blood test							
Hemoglobin, g/dL	13.4 (±1.6)	12.2 (±1.9)	<0.001	64.6	12.7 (±1.7)	12.4 (±1.9)	8.2
Creatinine, mg/dL	0.86 (±0.60)	1.05 (±1.12)	<0.001	21.6	0.99 (±1.03)	0.98 (±0.95)	0.9

Values are *n* (%), mean (±standardized difference), or median (interquartile range). ASD: absolute standardized mean difference.

**Table 3:** Mortalities according to days alive and out of hospital (DAOH) < 7.

	High Group	Low Group	HR (95% CI)	*p*-Value
Entire population	*n* = 4334	*n* = 1022		
One-year mortality	32 (0.7)	109 (10.7)	14.90 (10.05–22.10)	<0.001
Three-year mortality	67 (1.5)	168 (16.4)	10.71 (8.07–14.22)	<0.001
Five-year mortality	87 (2.0)	199 (19.5)	9.42 (7.32–12.12)	<0.001
Propensity-matched population	*n* = 864	*n* = 864		
One-year mortality	24 (2.8)	70 (8.1)	3.96 (2.24–6.99)	<0.001
Three-year mortality	45 (5.2)	112 (13.0)	3.82 (2.33–6.28)	<0.001
Five-year mortality	51 (5.9)	135 (15.6)	3.07 (2.04–4.61)	<0.001

HR: hazard ratio; CI: confidence interval.

**Table 4:** Subgroup analysis for association between one-year mortalities and days alive and out of hospital (DAOH) < 7.

	High Group	Low Group	HR (95% CI)	*p*-Value	*p* for Interaction
Age under 60	296 (50.4)	291 (49.6)	11.10 (2.61–47.21)	0.001	0.03
Age over 60	568 (49.8)	573 (50.2)	2.09 (1.25–3.49)	0.005	
Female	556 (50.0)	557 (50.0)	3.11 (1.63–5.93)	0.001	0.67
Male	308 (50.0)	307 (50.0)	5.58 (2.07–15.04)	0.001	
No general anesthesia	638 (50.4)	628 (49.6)	2.83 (1.47–5.45)	0.002	0.99
General anesthesia	226 (48.9)	236 (51.1)	4.08 (1.81–9.23)	0.001	
No total hip surgery	277 (48.9)	289 (51.1)	2.44 (1.47–4.05)	0.001	0.35
Total hip surgery	587 (50.5)	575 (49.5)	9.27 (1.76–48.76)	0.01	
No emergency surgery	497 (49.8)	704 (50.2)	4.69 (2.31–9.49)	<0.001	0.63
Emergency surgery	167 (51.1)	160 (48.9)	2.22 (1.02–4.85)	0.046	

HR: hazard ratio; CI: confidence interval.

## Data Availability

Available upon reasonable request.
